# Accelerated hypertension following mavacamten introduction in severe obstructive hypertrophic cardiomyopathy with hypertension: a case report

**DOI:** 10.1093/ehjcr/ytae450

**Published:** 2024-08-26

**Authors:** Niccolò Maurizi, Panagiotis Antiochos, Olivier Muller, Gregory Wuerzner, Pierre Monney

**Affiliations:** Service of Cardiology, Lausanne University Hospital, Rue de Bugnon 46, 1011 Lausanne, Switzerland; Service of Cardiology, Lausanne University Hospital, Rue de Bugnon 46, 1011 Lausanne, Switzerland; Service of Cardiology, Lausanne University Hospital, Rue de Bugnon 46, 1011 Lausanne, Switzerland; Service of Nephrology, Lausanne University Hospital, Rue de Bugnon 46, 1011 Lausanne, Switzerland; Service of Cardiology, Lausanne University Hospital, Rue de Bugnon 46, 1011 Lausanne, Switzerland

**Keywords:** Hypertrophic cardiomyopathy, Mavacamten, Hypertension, Case report

## Abstract

**Background:**

Mavacamten in Phase 2 and 3 clinical trials was well tolerated, reduced left ventricular outflow tract obstruction (LVOTO), and improved exercise capacity and symptoms. However, due to its recent introduction in the market, there is limited evidence from real-world patients with severe/multiple comorbidities and/or who are exposed to potential treatment interactions. Hypertension is common in patients with hypertrophic cardiomyopathy (HCM), but its impact on the treatment of LVOTO is undefined.

**Case summary:**

A 55-year-old man with severely obstructive symptomatic HCM and Grade I arterial hypertension underwent treatment with mavacamten 5 mg. He presented an accelerated hypertension from Day 10 of treatment. On admission, he reported improvement of his dyspnoea [New York Heart Association (NYHA) Class II] and NT-pro BNP decreased to 1646 ng/L. Echocardiography showed a left ventricular ejection fraction of 60% with reduced systolic anterior motion and LVOTO (max 21 mmHg). Causes of secondary hypertension were excluded, and blood pressure (BP) was controlled by eplerenone and amlodipine introduction. Accelerated hypertension was concluded as a final diagnosis, and a potential causal link with the introduction of mavacamten was made. Evolution up to Day 135 proved a stabilization of the BP profile and of the LVOT gradient (max 36 mmHg) as well as improvement in functional capacity (NYHA Class I).

**Discussion:**

We hypothesize that rapid relief of excess afterload may induce alterations potentially leading to high BP in patients with impaired peripheral vascular resistances. Patients with severe obstructive HCM and hypertension should be given special attention during mavacamten titration and should self-monitor the BP during this phase.

**Learning points**

- Given the limited real-world experience with myosin inhibitors and potentially comorbid patients, this case might suggest that specific caution should be given to patients with severe obstructive hypertrophic cardiomyopathy (HCM) and hypertension during the titration phase of mavacamten.
- Such patients should be encouraged to self-monitor their blood pressure during the titration phase, and an attempt to control baseline hypertension should be made before myosin inhibitor treatment initiation.
- A possible rapid relief of excess afterload may induce alterations potentially leading to high blood pressure in patients with impaired peripheral vascular resistances.
- Due to the lack of real-world case series, the causal relationship and the pathophysiological mechanism between myosin inhibitor initiation in severe obstructive HCM and hypertension should be confirmed by larger registry data.

## Introduction

Mavacamten is a first-in-class, targeted, cardiac-specific myosin inhibitor for the treatment of adults with symptomatic New York Heart Association (NYHA) Class II and III hypertrophic obstructive cardiomyopathy (HOCM).^1^ Developed to target the hyper-contractile phenotype pivotal in the disease's pathophysiology,^1^ mavacamten has shown in Phase 2 and 3 clinical trials a sustained reduction in left ventricular outflow tract (LVOT) gradients and improvement in exercise capacity and symptoms, while being well tolerated.^2,3^ However, real-world evidence in patients with severe comorbidities or potential treatment interactions remains limited due to its recent market introduction. Hypertension is common in patients with hypertrophic cardiomyopathy (HCM), but its impact on the treatment of LVOT obstruction is undefined.

## Summary figure

**Figure:**
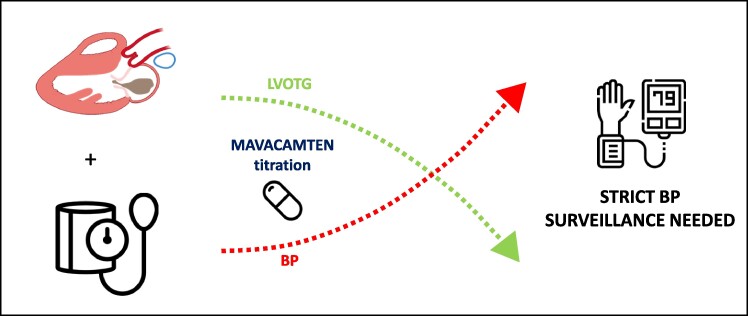
Accelerated hypertension after mavacamten introduction. BP, blood pressure; LVOTG, left ventricular outflow tract gradient.

## Case summary

**Figure 1:**
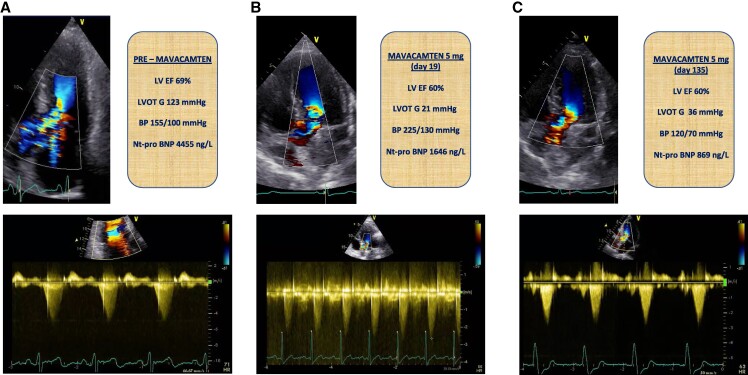
Serial echocardiographic evaluation pre mavacamten introduction, at 19 days of treatment and at 135 days. (*A*) shows bottom left apical view 5 chamber showing the sub-aortic acceleration with the associated mitral insufficiency, whereas below the continuous Doppler spectrum on the left ventricular outflow tract is showed, up to 123 mmHg. (*B*) presents bottom left apical view 5 chamber showing the reduction sub-aortic acceleration, whereas below the continuous Doppler spectrum on the left ventricular outflow tract shows no significant gradient at 21 mmHg. (*C*) shows bottom left apical view 5 chamber confirming the reduction of sub-aortic acceleration, and below the continuous Doppler spectrum on the left ventricular outflow tract shows sustained reduction of the gradient, at 36 mmHg. BP, blood pressure; LVEF, left ventricular ejection fraction; LVOTG, left ventricular outflow tract gradient.

Here, we present a case of a 55-year-old man, diagnosed with severe HOCM, who was referred to our clinic to evaluate the options for invasive or non-invasive management of the symptoms associated to his left LVOT gradient. He complained of dyspnoea NYHA Classes II–III associated with exertional and post-prandial light-headedness and pre-syncope. There was no history of non-sustained ventricular tachycardia, syncope, or family history of sudden death. The patient was sedentary and slightly overweight (28 kg/m^2^), but no other relevant comorbidities were present. He had Grade I arterial hypertension despite treatment with olmesartan 20 mg, hydrochlorothiazide 12.5 mg, and bisoprolol 10 mg. The electrocardiogram (ECG) showed sinus rhythm, with normal atrioventricular (PR 146 ms) and intraventricular conduction (QRS 112 ms). High QRS voltage and deep T-wave inversion associated with ST depression were present from V3 to V6. Blood pressure (BP) was 155/100 mmHg. Blood analysis showed a normal complete blood cell count, slightly reduced renal function with an estimated glomerular filtration rate (eGFR) of 59 mL/min/1.73 m^2^ without electrolyte abnormalities and a NT-pro BNP of 4455 ng/L. Physical examination showed a systolic ejection murmur, 3/6 at mitral and aortic spaces, increasing to 5/6 during Valsalva. Echocardiography showed a non-dilated left ventricle, with left ventricular hypertrophy (LVH) of the basal septum (max 23 mm vs. 12 mm in the posterior wall) with a left ventricular ejection fraction (LVEF) of 69% with no regional wall motion abnormalities but severe global longitudinal strain reduction (−9.9%); mitral valve presented a systolic anterior motion (SAM), responsible for a dynamic LVOT obstruction (58 mmHg at rest and 123 mmHg at Valsalva), with mild-to-moderate insufficiency. Grade II diastolic dysfunction was present, with an E/A ratio of 1.7, E/e′ of 22.6, and left atrial dilation (42 mm), and right ventricle was not dilated with normal longitudinal function (*Figure 1A*). A cardiac magnetic resonance imaging was performed, showing diffuse late gadolinium enhancement, specifically mid-wall inferior, basal lateral, and slightly circumferential at the apex without evidence of aneurysm. Extracellular volume at the level of the hypertrophied septum was 29.7%, with a T1 of 1046. Alpha-galactosidase activity was normal. Despite the maximal dose of beta-blockers, the patient was severely limited due to a severe LVOT gradient; therefore, 5 mg of mavacamten was started.

**Figure 2:**
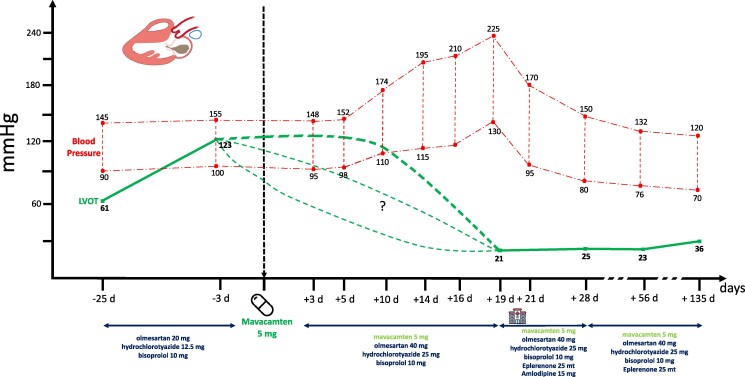
Evolution of self-monitored blood pressure and left ventricular outflow gradient before and during the mavacamten titration process. On *Y*-axis, blood pressure values are depicted, whereas on *X*-axis days, pre- and post-mavacamten introduction is shown. d, days; LVOT, left ventricular outflow tract.

On Day 10 of mavacamten therapy, the patient contacted our clinic because his BP was steadily rising, reaching 174/110 mmHg without any associated symptom or cause, and we increased the dose of olmesartan to 40 mg and hydrochlorothiazide to 25 mg. However, on Day 19 of mavacamten treatment, the patient was hospitalized because of an accelerated hypertension, with BP values up to 225/130 mmHg (*Figure 2*). On admission, he reported mild improvement of his dyspnoea (NYHA Class II) and the resolution of episodes of exertional and post-prandial light-headedness and pre-syncope. Blood pressure was 220/125 mmHg without asymmetry, and ECG showed no new changes. Chest radiography showed a normal cardiac silhouette, without signs of fluid overload. Laboratory analysis showed a normal complete blood cell count and normal renal function with EGFR > 60 mL/min/1.73 m^2^ without electrolytes or thyroid imbalances. Only a slight troponin T (TnT) increase was present (max TnT hs 19 mg/L) but NT-pro BNP decreased to 1646 ng/L. Serum and urine immunofixation were within normal limits. Cortisol and urinary metanephrines were normal. Echocardiography showed a non-dilated left ventricle, with an unchanged LVH pattern (max 23 mm) and an LVEF of 60% with no regional wall motion abnormalities. Mitral valve showed an important reduction in SAM, and LVOT obstruction was resolved (13 mmHg at rest and 21 mmHg at Valsalva). E/A ratio was 2, E/e′ was 20.2, and tricuspid insufficiency (TI) gradient was 52 mmHg. (*Figure 1B*). An abdominal ultrasound showed two normal-sized kidneys with no evidence of renal artery stenosis or surrenal adenoma. Rhythmic surveillance for 48 h did not show any significant arrhythmias. During the hospital stay, once secondary causes of hypertension were excluded, eplerenone 25 mg and amlodipine 15 mg were introduced, with a rapid improvement of the BP profile that led to discharge at Day 22 after mavacamten initiation. Because of the LVEF > 55% and important reduction in the LVOT gradient (21 mmHg), the 5 mg dose of mavacamten was continued. Accelerated hypertension resulting in hypertensive urgency was concluded as a final diagnosis, and a potential causal link with the introduction of mavacamten was made, as no other causes of secondary hypertension were identified.

Following intensification of anti-hypertensive treatment, the patient was seen 7 days after discharge. He confirmed the improvement in dyspnoea (NYHA Classes I and II) and the absence of episodes of exertional and post-prandial light-headedness and pre-syncope. Blood pressure was 150/80 mmHg with a low-to-normal home self-monitoring profile reported and minimal orthostatic hypotension. ECG was comparable and the echocardiography showed LVEF stable at 60% and minimal SAM without LVOT obstruction (25 mmHg at Valsalva). The right ventricle was not dilated with normal longitudinal function. E/A was 1.2, E/e′ normalized to 10.3, and the TI gradient was 31 mmHg. Given the normal home monitoring profile, amlodipine was discontinued. The evolution of the BP profile was optimal with 85% of the self-reported measures < 140/90 mmHg (*Figure 2*). The two successive follow-up visits, at Days 56 and 135 after the start of mavacamten treatment, confirmed stable LVEF (60%) with minimal SAM and LVOT obstruction at Valsalva (36 mmHg). An improvement in the diastolic function was confirmed (E/A ratio was 1.2, E/e′ normalized to 8.9, and the TI gradient was 31 mmHg) (*Figure 1C*).

## Discussion

Rapid relief of excess afterload may lead to alterations in systolic and diastolic function, ventriculo-arterial coupling, coronary blood flow, and, consequently, cardiac output, which can lead to hypertension in patients with altered peripheral vascular resistances.^4,5^ In our case, normalization of the severe dynamic LVOT gradient occurred after about 3 weeks of treatment with mavacamten, and resting self-monitored BP gradually increased to severe hypertension. Following the introduction of a myosin inhibitor, a reduction of actin–myosin cross-bridge formation occurs, along with a decreased contractility and an improvement in myocardial energetics.^6^ Concomitantly, due to the rapid decrease in LVOT obstruction, rapid changes in excess pressures could have occurred, thus, likely representing changes in the flow patterns and physiology that would potentially facilitate the up-regulation of the effective stroke volume with increasing physiological demand. Such mechanisms, in patient with increased arterial stiffness due to chronic hypertension, could lead to an accelerated hypertension. A similar phenomenon may be observed after septal myectomy in hypertensive patients and is well described after trans-catheter aortic valve replacement, although the pathophysiological substrate is different.^4,5^ In a *post hoc* analysis by Wang *et al*.^7^ of the Explorer-HCM study, the 60 hypertensive patients treated with mavacamten had significantly higher BP than those treated with placebo (130 ± 14.8 vs. 125 ± 15.3 mmHg; *P* < 0.01). However, no details were given concerning the nature of serious adverse events or the occurrence of hypertensive crisis. Moreover, patients treated in the SEQUOIA-HCM trial with aficamten, a myosin inhibitor with a more shallow dose–response relationship with respect to mavacamten, presented more hypertension with respect to placebo (7.7% vs. 2.1%),^8^ potentially suggesting a class effect.

Despite the lack of definitive evidence, we hypothesized that the accelerated hypertension was related to the introduction of the mavacamten treatment in a context of insufficient hypertension control. This aspect constitutes a still unmet clinical need: control of hypertension in severely HOCM is of potential concern, since most effective drugs are vasodilating and to be potentially avoided in such cases. We postulate that probably, the only available options in addition to beta-blockers are centrally acting anti-hypertensive drugs, such as moxonidine or methyldopa.

Lastly, we recognize that further cases would be needed to establish a definitive causal relationship with mavacamten and to elucidate the pathophysiological process. However, few clinically relevant points can be drawn from this case: (i) patients with severe obstructive HCM and hypertension should be given special attention during mavacamten titration; (ii) these patients should self-monitor the BP during this phase; and (iii) ideally, baseline hypertension should be treated and controlled the best way possible before initiation of myosin inhibitor treatment. Due to the lack of real-world case series, our observations need to be confirmed in larger registries to ascertain the causal relationship and the pathophysiological mechanism encompassing hypertension after myosin inhibitor initiation in severe obstructive HCM.

## Lead author biography


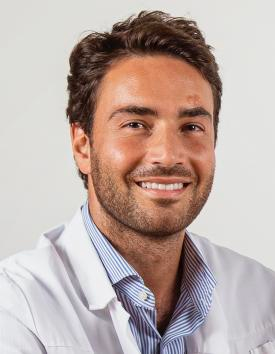


Niccolò Maurizi is a chief resident in Cardiology at CHUV|Lausanne University. Prior to that, he worked as a researcher at Università degli Studi di Firenze. He co-founded D-Heart, a start-up for innovative cardiologic wearable concepts in 2015. He has authored and co-authored almost 100 scientific articles with concentration on HCM-related issues (sudden cardiac arrest management, pregnancy in HCM, smart devices in clinical practice, etc.).

**Consent:** The patient gave his informed consent, in line with COPE guidelines for the present manuscript.

**Funding:** None declared.

## Data Availability

Data would be available anonymized upon reasonable request.
